# Changes in satisfaction and anxiety about radiotherapy for pediatric cancer by two-step audio-visual instruction

**DOI:** 10.1016/j.tipsro.2023.100214

**Published:** 2023-07-10

**Authors:** Hiroshi Fuji, Tomoyasu Fujibuchi, Hideyuki Tanaka, Yuu Ogawa, Chihiro Noda, Maoko Hayakawa, Kazuaki Nakamura, Kyoko Tanaka

**Affiliations:** a Division Of Radiation Oncology, National Center For Child Health And Development, Tokyo, Japan; b Division Of Psychosocial Medicine, National Center For Child Health And Development, Tokyo, Japan; c Department of Pharmacology, National Center for Child Health and Development, Tokyo, Japan

**Keywords:** Radiation oncology, Parents, Anxiety, Pediatrics, Instruction

## Abstract

**Objectives:**

Although radiotherapy is an essential component of pediatric cancer treatment, inadequate radiotherapy information for childhood cancer and unusual treatment situations can negatively affect parental perceptions and emotions. This study aims to investigate the effect of two-step audio-visual instruction system effects introduced by our institution on parent satisfaction and anxiety when initiating radiotherapy.

**Methods:**

The two-step audio-visual instruction system comprised instructive animation using patient avatars and a live video system. The live video system has a 55-inch-wide monitor, and a no-latency sound module. Parents in the radiotherapy division can view the patient in the treatment room through the live video system. This prospective study compared satisfaction and anxiety about radiotherapy introduction before and after two-step audio-visual instruction. We enrolled 20 parents whose child underwent radiotherapy, and they completed a set of questionnaires—Client Satisfaction Questionnaire, State-Trait Anxiety Inventory, and original questionnaires about radiotherapy.

**Results:**

Satisfaction scores improved significantly after two-step audio visual instruction (25.5 ± 3.4) compared with those before the instruction (27.7 ± 3.1) (p = <0.01). Anxiety scores also decreased significantly after the instruction (50 ± 9) compared with those before the instruction (54 ± 11) (p = 0.004). However, anxiety-related personality trait scores did not change drastically before and after viewing (48 ± 8.5 vs. 49 ± 7.5) (p = 0.419).

**Conclusion:**

This single-arm prospective study indicates that two-step audio-visual instruction for radiotherapy is effective in improving parents' anxiety about radiotherapy introductions. However, large-scale and comparative studies are warranted to generalize the benefit of two-step audio visual instruction.

## Highlights

• Inadequate radiotherapy information for childhood cancer can make parents develop negative perceptions and emotions.
• Audio-visual instruction system with instructive animation and live video system was used for the instruction of radiotherapy for parents.
• The audio-visual instruction is effective in improving parents' anxiety and satisfaction about radiotherapy.

## Introduction

Radiotherapy is the cornerstone treatment for pediatric cancers. It is introduced for definitive or adjuvant treatment of solid tumors and myeloablative arms ahead of stem cell transplantation in hematological malignancy treatment. Radiotherapy is painless and accompanied by fewer symptoms than other oncological treatments; however, the procedure and unique circumstances may cause distress among children undergoing radiotherapy [1], [2], [3], [4], [5], [6]. Therefore, children are often reluctant to undergo radiotherapy when they entered the preparation process. Particularly, immobilization techniques for the precise delivery of radiation beams are known to be uncomfortable for children. Similarly, face masks for the treatment of head and neck tumors are known to cause fear or discomfort, even in adulthood [7]. Once the procedure commences, presenting large machines and sounds in the treatment room and being left alone also leads to distress [1], [2], [3], [4], [5], [6], [8]. Therefore, younger children are occasionally treated with general anesthesia. However, general anesthesia is known to induce anticipation anxiety [1], [2], and fasting to prepare for anesthesia would make them uncomfortable for radiotherapy. Generally, radiotherapy is delivered daily in multiple fractions. Concerns regarding patient-treatment torelance and safety usually continue throughout the 2–5 treatment weeks, often causing continuous distress among their parents.

Additionally, deciding whether to use radiation for their children causes parental distress. They may have been provided with a various information on the pros and cons of radiotherapy when they provided consent. Long-term sequelae are one of the critical deficits for accepting radiotherapy. Nonetheless, most patients and parents feel that the mechanism of adverse events of radiotherapy is more difficult to understand than that of surgery or drugs [8].

Considering the difficulties and significance of the perception of radiotherapy in parents of pediatric patients with cancer, effective and appropriate instructions must be explored for parents as well as for the patients themselves [2], [3], [8].

At a radiation oncology department in a children's cancer center, we introduced a live video presentation system consisting of a wide and high-resolution screen for watching the radiotherapy process of patients out of the treatment room. It is mainly used for parents to watch their child's treatment in an open-style treatment delivery method. After several months of live video utilization, we implemented an introductory animation video to assist in comprehending radiotherapy procedure before watching the live videos. Eventually, a two-step audio-visual instruction system combined a live video and instruction animation.

This study aims to evaluate the impact of these tools on the anxiety and satisfaction of parents whose children received radiotherapy. In addition, to characterize the emotions of parents with respect to the instructional system, factors contributing to parents' anxiety and satisfaction before and after instruction were also investigated.

## Materials and methods

### Participants

This single cohort study was conducted between June 2021 and June 2022 after approval by the internal review board of the public children's hospital. Written informed consent was obtained from all participants. The two-step audio-visual instruction was only available for individuals allowed to stay in the radiation oncology department under infection control restrictions at the children hospital. Parents of children who received radiotherapy and could use two-step audio visual instruction were approached for enrollment in the study.

### Audio-visual intervention

Two-step audio-visual instructions with introductory animation and live video for treatment followed by animation were provided after obtaining informed consent.

### Introductory animation

Introductory animations were played to facilitate an understanding of the treatment process and circumstances before watching the live videos. The animation, with a length of 3–6 min, consists of an avatar character for a virtual patient and a treatment room photograph as a background scene. In the animation, an avatar child acts as a patient, and an avatar doctor explains each step of the treatment and devices. Some of the animations also included a demonstration of the treatment mechanism and setting up.

### Telepresence system

A set of telepresence systems, originally developed for real-time personal communication systems MADO^TM^ (SONY, Tokyo, Japan), was introduced to the radiation oncology department. Two terminals were separately placed in the treatment room and at the reception area of the radiation oncology department. Both computer terminals were connected to a resolution 55-inch monitor and a high-resolution web camera. For close monitoring of the treatment procedure and patient behavior during treatment, a camera in the treatment room was installed using arms hanging from the ceiling of the treatment room. Notably, this telepresence system adopts a unique sound system for live communication. Sound adaptation systems have less latency and prioritize human voice to enhance natural communication, even over the internet.

### Procedure

The introduction of radiotherapy to both parents and children with cancer was provided in the usual manner to obtain informed consent for radiotherapy. After obtaining informed consent, demographic data were collected from electronic charts. The survey measurements were collected after obtaining informed consent　and watching both videos. The introductory animation and live video impact were evaluated separately. The same questionnaire set was used in each evaluation process.

One of the two questionnaires used was the State-Trait Anxiety Inventory (STAI) measuring two participant anxiety domains [9]. One is the state, mood, or temporary emotions related to anxiety. The other is the trait, personal features, or temperament inclined to have anxiety. The STAI comprises 22 questions, and higher inventory scores represent more anxiety.

**Fig. 1:**
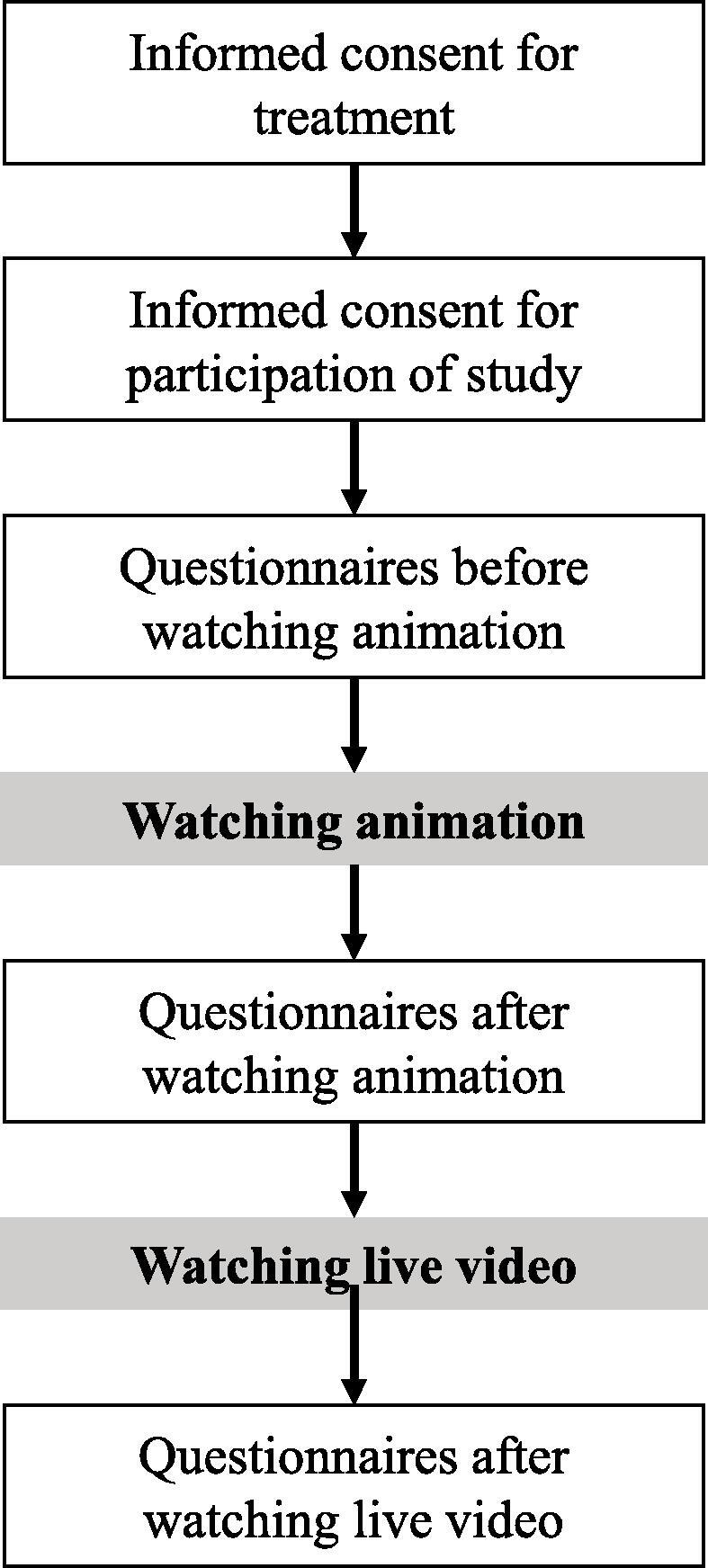
Flow diagram of two-step audio-visual instruction.

The second test was the client satisfaction questionnaire (CSQ-8), commonly used for medical service evaluation [10]. It included eight questions on a Likert scale. Each question represents satisfaction extent and their sum gives the total score. A higher score indicates higher satisfaction. The flowchart of this study is shown in Fig. 1.

### Statistics

The collected data were analyzed using the R statistical software (version 4.1.2: R Core Team. R Foundation for Statistical Computing, Vienna, Autstria). The differences in STAI and CSQ-8 scores after instructions were tested using a paired *t*-test. The relationship between STAI and CSQ-8 scores obtained at each point was analyzed using Pearson correlation. The dependency of participants' backgrounds on these scores was examined using an independent sample *t*-test.

## Results

### Characteristics of participants

All parents who could watch the radiotherapy of their children signed their consent to participate in this study; among them, 19 eventually enrolled in this study. The demographic data of the participants are presented in Table 1. Both mothers (n = 13) and fathers (n = 6) were included in this study. The mean age of patients who underwent radiotherapy was 7.5 years, and their diseases included hematological disease (n = 6), and olid tumors (n = 13).

**Table 1:** Participants’ characteristic.

Characteristic	Category	n
Gender of participant	female	13
	Male	6
Gender of patient (child)	Female	6
	Male	13
Type of disease	Hematological	6
	Solid	13
Age of patient (child)	<7.5	11
	≥7.5	8
Anesthesia	Applied	3
	Not applied	16

### Changes in anxiety score

**Fig. 2:**
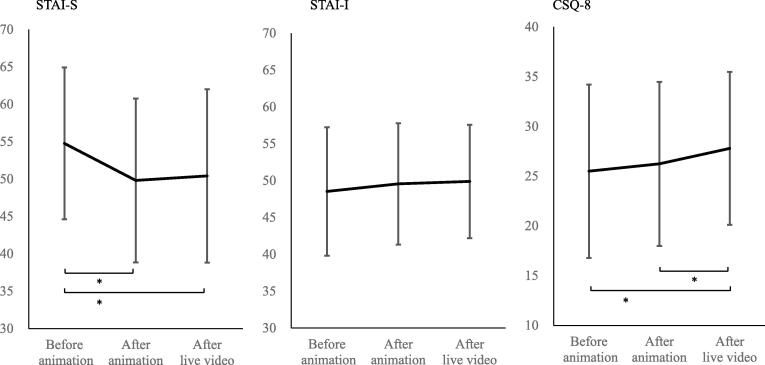
Changes in psychological scores by two-step audio-visual instruction. *: p < 0.05.

The measured STAI scores for each step are shown in Fig. 2. The STAI-State score before the presentation was 54.8. This score decreased significantly after watching the animation (49.8) but did not change after watching live videos (54.8). Notably, the STAI-State score after the live video telecast was significantly lower than the background score (p = 0.005), whereas STAI-Trait scores did not change significantly They were consistently approximately 49, without any significant differences.

### Changes in satisfaction score

The satisfaction score of the parents at the beginning of the study and after watching the introductory animation were 25.5 and 26.2, respectively (Fig. 2.). After watching the live video, this increased to 27.7, at a statistically significant difference (p = 0.025).

### Factors affecting anxiety and satisfaction

The relationship between anxiety and satisfaction scores was generally negative. Only scores measured after watching the live video revealed statistically significant relationships. There was no remarkable difference in the STAI scores between the stratified groups (Table 2). Significant differences between older and younger age groups (p = 0.042). and male and female parents (p = 0.013) were observed in the CSQ-8 scores measured before watching the animation (Table 3).

**Table 2:** Influence of participant characteristics on anxiety scores measured before and after intervention.

Note: STAI = State-Trait-Anxiety Inventory.

**Table 3:** Influence of participant characteristics on satisfaction scores measured before and after intervention.

Note: CSQ-8 = Client Satisfactory Questionnaire-8; t = *t*-test for paired groups. * Statistically significant at P < 0.05.

## Discussions

The parents of children with serious diseases are generally stressed about treatment decision-making, concerns for their children's future, and incorporated them into treatment plans [11], [12], [13]. Therefore, controlling the emotional status of parents of children with severe diseases is important for the well-being of patients' families [14]. European groups reported that the period of radiotherapy treatment for children could be a significant source of distress for parents of children scheduled for radiotherapy. This applies to parents those whose children underwent surgery or planned to undergo surgery by themselves [2], [3], [4], [13], [15]. Upon comparing the score obtained as background in the current study with those obtained in a previous Japanese study that measured female patients’ anxiety, the anxiety level of parents of children was comparable to or higher than those of female patients themselves [16].

Several reports have investigated children's distress in medical procedures and the efficiency of instructions. However, the benefit of these instructions is not sufficient to apply to children, and the reported indications for the instructions are limited age and acceptability [1], [2], [5]. These studies also disclosed that parental anxiety is one of the factors contributing to children’s anxiety. This present study demonstrateds that two-step audio-visual instructions for parents decreases their anxiety at the beginning of radiotherapy. Therefore, we consider that this system might be a clue to developing instructions for radiotherapy as a tool for improving the well-being of patients and patients' families.

The current study investigating parents' anxiety through two-step audio-visual instruction showed the impacts of the early part of instruction utilizing introductory animation video and the later part of instruction utilizing live video separately. A decrease in STAI-S scores after watching the introductory animation was found to be significant, but the changes in STAI-S scores after watching the live video were not significant. The lower impact of the latter part of the two-step audio-visual instruction on parents' anxiety may be attributed to the timing of anxiety measurement rather than the efficiency of the intervention. Alterations in anxiety levels have been reported in patients waiting for medical procedures, such as those undergoing surgery, anesthesia, and radiotherapy [1], [17].

An increase in satisfaction through two-step audio-visual instruction was revealed by the CSQ-8, which is generally used in the measurement of medical service satisfaction in adults. Many studies on medical procedure instruction have implemented this score and demonstrated the efficiency of the intervention to improve adult patients’ satisfaction [18], [19], [20]. Few reports on interventions for children or parents of sick children have shown a minimal impact on the satisfaction of parents [15], [21], [22]. A study utilizing a unique score for parents of sick children showed an improvement in satisfaction with education for parents. Although utilizing the CSQ-8 precludes the comparison of the satisfaction level in those early studies for parents, it allows us to compare the significance of our intervention with other interventions based on satisfaction-specific scoring.

The characterization of the population that is effective for instruction or needs instruction is another theme for developing instructional tools. To date, no report has predicted a more effective population for any type of instruction for parents of children undergoing radiotherapy. The current study predicted the relationship between satisfaction and anxiety in parents of children undergoing radiotherapy. Female parents tended to be more satisfied after watching a video. Thus, we believe that these demographic analysis data and comparative analysis of different psychological indexes will provide clues for exploring the mechanism of moderating parents’ emotions and establishing the adaptation of audio-visual intervention.

This study had several limitations. The first limitation is that single-group analysis was used. The current study demonstrated the impact of two-step audio-visual information by comparing the data collected before and after the intervention. This study, with sequential assessment for a single group, harbors concern regarding the effects of confounders, especially regarding the timing of assessment.

The second limitation is the convenient sample size. Considering the feasibility of the study and the limited information we had about the interventions and participants; the sample size of this study is only helpful in establishing the study's design in future studies.

The third limitation is that the combination of audio-visual content in the two-step instruction makes the role of each intervention unclear. At the time of launching this study, we expected that a set of introductory animation and live videos would maximize the benefits for parents' families. For those limitations in study design, crossover design study evaluating each intervention separately while ensuring they provide equal benefit to the participant will be warranted.

## Conclusions

We found that two-step audio-visual instruction is feasible for parents of children undergoing radiotherapy. Even though a previous report predicted that audio-visual instruction has a limited impact on parents of children undergoing radiotherapy, the promising positive impact of two-step audio-visual instruction on parents' emotions may be a clue to exploring techniques to relieve parents' distress shown during the radiotherapy procedure of their children. In addition, information about the relationship between each emotional level and demographic data predicted that large-scale multifactorial analysis of the parents applying this instruction might be a promising way to explore parents' emotions and their contribution to child well-being.

Funding

This work was supported by the Council for Science, Technology and Innovation (CSTI), Cross-ministerial Strategic Innovation Promotion Program (SIP), “Innovative AI Hospital System” (Funding Agency: National Institute of Biomedical Innovation, Health and Nutrition (NIBIOHN)).

## Declaration of Competing Interest

The authors declare that they have no known competing financial interests or personal relationships that could have appeared to influence the work reported in this paper.
